# Glucose Regulation of Thrombospondin and Its Role in the Modulation of Smooth Muscle Cell Proliferation

**DOI:** 10.1155/2010/617052

**Published:** 2010-06-20

**Authors:** Laura A. Maile, Lee B. Allen, Christopher F. Hanzaker, Katherine A. Gollahon, Paul Dunbar, David R. Clemmons

**Affiliations:** Division of Endocrinology, University of North Carolina, CB# 7170, 5030 Burnett Womack, Chapel Hill, NC 27599-7170, USA

## Abstract

Smooth muscle cells (SMC) maintained in high glucose are more responsive to IGF-I than those in normal glucose. There is significantly more thrombospondin-1 (TSP-1) in extracellular matrix surrounding SMC grown in 25 mM glucose. In this study we investigated 1) the mechanism by which glucose regulates TSP-1 levels and 2) the mechanism by which TS-1 enhances IGF-I signaling. The addition of TSP-1 to primary SMC was sufficient to enhance IGF-I responsiveness in normal glucose. Reducing TSP-1 protein levels inhibited IGF-I signaling in SMC maintained in high glucose. We determined that TSP-1 protected IAP/CD47 from cleavage and thereby facilitated its association with SHP substrate-1 (SHPS-1). We have shown previously that the hyperglycemia induced protection of IAP from cleavage is an important component of the ability of hyperglycemia to enhance IGF-I signaling. Furthermore we determined that TSP-1 also enhanced phosphorylation of the *β*3 subunit of the *α*V*β*3 integrin, another molecular event that we have shown are critical for SMC response to IGF-I in high glucose. Our studies also revealed that the difference in the amount of TSP-1 in the two different glucose conditions was due, at least in part, to a difference in the cellular uptake and degradation of TSP-1.

## 1. Introduction

Atherosclerosis occurs more frequently in patients in which the vascular environment has been disrupted due to changes in the metabolic (e.g., diabetes) or mechanical (e.g., hypertension) environment. Insulin-like growth factor-I (IGF-I) stimulates smooth muscle cell (SMC) proliferation and has been implicated in the lesion progression [1–3]. When SMCs are grown in hyperglycemic conditions, they are more responsive to IGF-I than those grown in normo-glycemic conditions. We have determined that, under these conditions, the enhanced proliferative response of SMC to IGF-I is dependent upon two variables. First, the significant increase in the amount of three *α*V*β*3 ligands including TSP-1, osteopontin, and vitronectin (Vn) within the extracellular matrix (ECM) surrounding SMC grown in 25 mM glucose compared with those grown in 5 mM [4]. A second important variable is the increase in association between the extracellular domains of CD47/IAP (integrin associated protein) and SHP substrate 1 (SHPS-1) which is required for IGF-I responsiveness of SMC [5, 6]. The increase in association between the two proteins is due to an increase in intact CD47/IAP as a result of hyperglycemia induced protection from matrix-metalloprotease-2 (MMP-2) mediated cleavage [6].

TSP-1 is a ligand for CD47/IAP [7] and enhances IGF-I signaling in SMC [8]. When aortic extracts from hyperglycemic mice were compared with normo-glycemic mice there was a significant increase in TSP-1 and TSP-1 binding to CD47/IAP [9]. Associated with this was an enhanced proliferative response to IGF-I. These current studies were undertaken to determine how TSP-1 functions to modulate the SMC response to IGF-I and the mechanisms by which hyperglycemia regulates TSP-1 protein.

## 2. Experimental Procedures

### 2.1. Details Provided in Supplemental Information 

#### 2.1.1. Porcine SMC (SMC)

SMCs were isolated and maintained as we have described previously [4, 6].

#### 2.1.2. Preparation of Aorta from Normal and Hyperglycemic Pigs

Male Yorkshire pigs (*n* = 8) were purchased from Clemson University. Animals that were 2 months of age were utilized. They were maintained according to the Guide for care of laboratory animals (NIH publication #85-23). Pigs on a high fat diet were infused with streptozotocin (STZ: 50 mg/kg/day) for 3 days. Mean fasting blood glucose rose from 81 ± 11 to 360 ± 123 mg/dL 7 days after the infusion. Insulin (27 ± 12 Units) was administered daily to the diabetic animals. Control animals gained 112 ± 5 lbs and hyperglycemic animals gained 77.5 ± 8 lbs during the course of the study. Three and a half months later the animals were euthanized. Femoral arteries were collected and homogenized in modified RIPA [10].

#### 2.1.3. RNA Interference (RNAi)

Lentivirus containing the pLenti6/BLOCK-iT DEST vectors + TSP-1 or scrambled control RNAi constructs was produced in 293FT cells and SMCs were transduced [11]. Following selection, (growth medium containing 4 *μ*g/mL blasticidin) the cultures were grown to confluency and reduction in TSP-1 protein confirmed by western immunoblotting.

#### 2.1.4. Cell Proliferation

The increase in cell number stimulated by IGF-I was determined as we have described previously [4].

#### 2.1.5. Cell Treatments

SMCs were plated in high (25 mM; H) or normal (5 mM; N)-growth medium (GM) and grown for 24–120 hours. Confluent monolayers were rinsed three times with H or N-serum-free medium (SFM) and incubated overnight (16-17 hours) in SFM prior to the addition of inhibitors, vehicle peptides and/or IGF-I (50 ng/mL). Bafilomycin A and Cathepsin inhibitor I were reconstituted in ethanol and used at 100 nM. TSP-1, 4N1K, and 4NGG were prepared in phosphate buffered saline (PBS) and used at 1 *μ*g/mL.

#### 2.1.6. Extracellular Matrix (ECM) Isolation

ECM was prepared as we have described previously [12].

#### 2.1.7. Cell Membrane Preparation

Cell membranes were prepared as we have described previously [13].

#### 2.1.8. Real-Time PCR Analysis of TS-1 mRNA

Total RNA was harvested using the Qiagen RNAeasy kit. cDNA was made using 1 *μ*g of RNA (High Capacity cDNA Reverse Transcription Kit; Applied Biosystems Foster City, CA). Real-Time PCR reactions were set up using the TaqMan Gene Assay kit and porcine TSP-1 (ABI Catalog number: SS03373876_m1 THBS1) primer probe sets. Reactions were performed in quadruplicate. ABI SDS 2.2.2 software was used to determine relative amounts of RNA.

#### 2.1.9. Protein Estimation and Visualization

Protein concentrations were determined using a BCA protein assay kit (Pierce, Rockford IL). All samples were loaded on the gel at the same concentration of total protein. Proteins were visualized using immunoblotting as we have previously described [8].

#### 2.1.10. Statistical Analysis

Chemiluminescent images obtained were scanned using a DuoScan T1200 (AGFA Brussels, Belgium) and band intensities of the scanned images were analyzed using NIH Image, version 1.61. The Student's *t* test was used to compare differences between treatments. The results that are shown in all experiments are representative of at least three separate experiments.

## 3. Results

### 3.1. Correlation between TSP-1 Levels and Levels of Intact IAP

**Figure 1:**
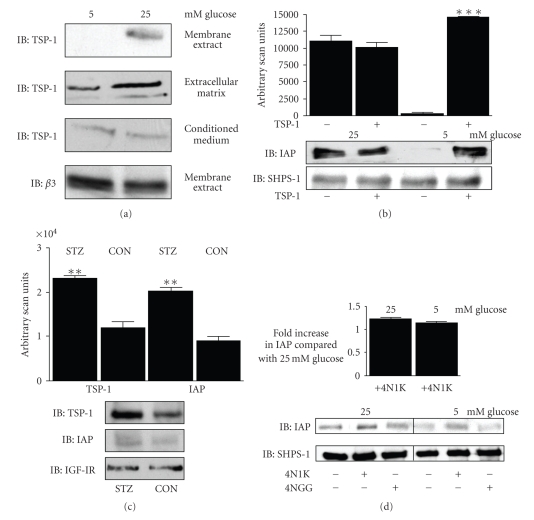
TSP-1 levels correlate with the level of intact IAP in SMC. (a) SMC were grown to confluency in DMEM containing 5 or 25 mM glucose and then incubated overnight in SFM with the appropriate level of glucose. Extracellular matrix, membrane extract or conditioned medium was harvested as described in the methods. Equal amounts of protein were immunoblotted (IB) with either an anti-TSP-1 or *β*3 antibody. (b) SMC were grown and incubated overnight in SFM as described for (a). The following day the incubation was continued for a further 6 hours after the addition (+) of TSP-1, (1 *μ*g/mL). The graphs show arbitrary scanning units derived from the immunoblots with the anti-IAP antibody from three independent experiments. ***P < .005 when the level of IAP in the presence of TSP-1 is compared to SFM alone. (c) Equal amount of protein from the homogenates of aorta from normal (Con) and hyperglycemic (STZ) pigs were separated by SDS PAGE and the level of TSP-1, IAP and the IGF-IR determined by western immunoblotting (IB). The graphs shows show arbitrary scanning units derived from the immunoblots with the anti-TSP-1 or IAP antibody, as labeled, from three independent experiments. **P < .01 when the level of TSP-1/IAP from the STZ animals is compared with control. (d) SMC were grown and incubated overnight in SFM as described for (a). The following day the incubation was continued for a further 6 hours after the addition (+) of 4N1K or 4N1G, (1 *μ*g/mL). The graph shows the fold difference in IAP levels compared with the level derived from SMC incubated in 25 mM glucose using arbitrary scanning units from the immunoblots with the anti-IAP antibody from three independent experiments.

Consistent with our previous observation [4] there was significantly more (14 ± 1.6 fold [mean ± SEM, *n* = 3, *P* < .005]) TSP-1 associated with the cell membrane of SMC maintained in high glucose conditions compared with those maintained in normal (Figure 1(a) top panel). We determined that there was significantly more TSP-1 (3.7 ± 0.  7 fold [mean ± SEM, *n* = 3, *P* < .005]) deposited in the ECM of SMC maintained in medium containing high compared with normal glucose (Figure 1(a) second panel). There was no significant difference in the amount of TSP-1 that was detected in the conditioned medium (Figure 1(a) third panel).

Since TSP-1 is a ligand for CD47/IAP [7] we determined whether the lack of TSP-1 in normal glucose conditions may play a role in the proteolysis of CD47/IAP by adding TSP-1 to SMC maintained in normal glucose. Using an antibody that specifically detects the intact, but not proteolytically cleaved CD47/IAP [6], there was significantly less intact IAP in SMC maintained in normal glucose compared with high. Addition of TSP-1 resulted in a significant increase in the amount of intact CD47/IAP that could be detected in SMC maintained in normal glucose, such that the level of intact CD47/IAP was equivalent to that seen in SMC maintained in high glucose (Figure 1(b)). 

To demonstrate that a similar increase in TSP-1 and CD47/IAP levels occurs in vivo in response to hyperglycemia we examined the amount of TSP-1 and IAP in aorta homogenates from hyperglycemic and control pigs. Consistent with our previous findings in mice [9] we determined that there was a significant (2 ± 0.1 fold [mean ± SEM, *n* = 3, *P* < .005]) increase in the amount of TSP-1 that was present in the aorta from the hyperglycemic pigs. This was associated with a significant (2.1 ± 0.1 [mean ± SEM, *n* = 3, *P* < .005]) increase in the amount of intact CD47/IAP that could be detected (Figure 1(c)). There was no significant difference in the amount of IGF-I receptor (IGF-IR). While the aorta homogenates contain a mixture of cells including, SMC, the data supports our in vitro findings that hyperglycemia is associated with an increase in TSP-1 and CD47/IAP.

To determine whether TSP-1 binding to CD47/IAP played a role in the increase in intact CD47/IAP, we examined the effect of small synthetic peptide (4N1K) homologous to the CD47/IAP binding site of TSP-1 and a control peptide 4NGG [8]. Addition of 4N1K but not 4NGG resulted in a significant (10 ± [mean ± SEM, *n* = 3, *P* < .005]) increase in the amount of intact CD47/IAP that could be detected in SMC maintained in normal glucose (Figure 1(d)) such that the amount of intact CD47/IAP was equivalent to that seen in SMC cultured in high glucose as shown in the graph in Figure 1(d).

### 3.2. Addition of the IAP Binding Site of TSP-1 Enhances IGF-I Signaling in SMC in Normal Glucose

CD47/IAP binding to SHPS-1 is a prerequisite for IGF-I stimulated SHPS-1 phosphorylation which in turn is required to recruit and facilitate the phosphorylation of the adaptor protein Shc [11] and downstream signaling pathways [11, 14] leading to increases in cell proliferation and migration [15]. 

**Figure 2:**
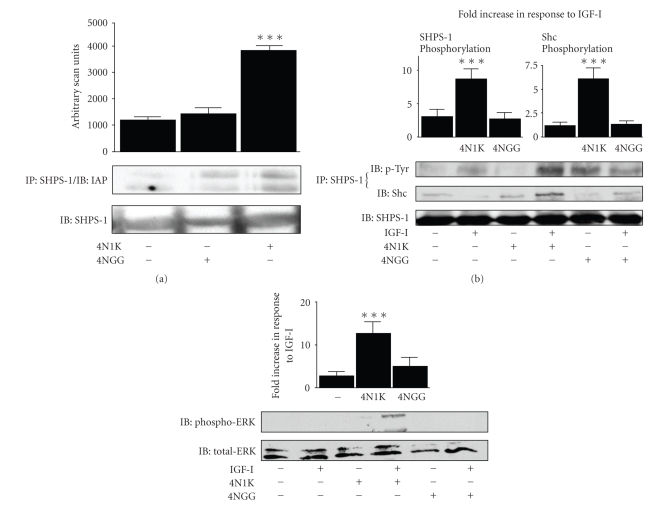
The IAP binding domain of TSP-1 increases IGF-I signaling in normal glucose. (a) SMCs were grown to confluency in DMEM containing 5 mM glucose and then incubated overnight in SFM with 5 mM glucose. The following day the incubation was continued for 6 hours with (+) 4N1K or 4NG1G (1 *μ*g/mL). Equal amounts of lysate were then subjected to immunoprecipitation (IP) with and anti-SHPS-1 antibody prior to immunoblotting (IB) with an anti-IAP antibody or immunoblotted directly with an anti-SHPS-1 antibody. The graph shows the results from three similar experiments expressed as arbitrary scanning units (***P < .005 when the amount of IAP associated with SHPS-1 is compared with the value for SMC incubated in 5 mM glucose containing medium). (b) SMCs were grown to confluency in DMEM containing 5 mM glucose and then incubated overnight in SFM with 5 mM glucose. The following day the incubation was continued for 6 hours with (+) 4N1K or 4NG1G (1 *μ*g/mL) followed by IGF-I (50 ng/mL) for 5 minutes. Equal amounts of lysate were then subjected to immunoprecipitation (IP) with and anti-SHPS-1 antibody prior to immunoblotting (IB) with either an antiphosphotyrosine (p-Tyr) or Shc antibody or immunoblotted directly with an anti-SHPS-1 antibody. The graph shows the results from three similar experiments expressed as fold increase in response to IGF-I compared with no addition of peptide (***P < .005). (c) SMCs were grown to confluency in DMEM containing 5 mM glucose and then incubated overnight in SFM with 5 mM glucose. The following day the incubation was continued for 6 hours with (+) 4N1K or 4NG1G (1 *μ*g/mL) followed by IGF-I (50 ng/mL) for 5 minutes. Equal amounts of lysate were then subjected to immunoblotting (IB) with either an anti-phosphoERK or total-ERK antibody. The graph shows the results from three similar experiments expressed as fold increase in response to IGF-I compared with no addition of peptide (***P < .005).

Addition of 4N1K, but not 4NGG, to SMC maintained in normal glucose containing medium resulted in a significant (3.3 ± 0.1 fold [mean ± SEM, *n* = 3, *P* < .005]) increase in CD47/IAP association with SHPS-1 (Figure 2(a)). In normal glucose conditions IGF-I cannot stimulate an increase in SHPS-1 phosphorylation [6]. Following treatment with 4N1K there was a significant (8 ± 1.4 fold [mean ± SEM, *n* = 3, *P* < .005]) increase in SHPS-1 phosphorylation and a significant (6.2 ± 1.1 [mean ± SEM, *n* = 3, *P* < .005]) fold increase in Shc phosphorylation in response to IGF-I (Figure 2(b)). Addition of 4NGG had no effect. 

In some experiments, we observe a basal increase in SHPS-1 phosphorylation however, it is not associated with any increase in Shc phosphorylation or downstream signaling (Figure 2(b)) presumably due to the lack of increase in SHPS-1 phosphorylation in response to IGF-I.

There was a significant (12 ± 3 fold [mean ± SEM, *n* = 3, *P* < .005]) increase in pERK1/2 phosphorylation in response to IGF-I which we have shown is required for IGF-I stimulated SMC proliferation (Figure 2(c)).

### 3.3. Reducing TS-1 Protein Using RNA Interference (RNAi) Decreases Intact IAP and Inhibits IGF-I Signaling

**Figure 3:**
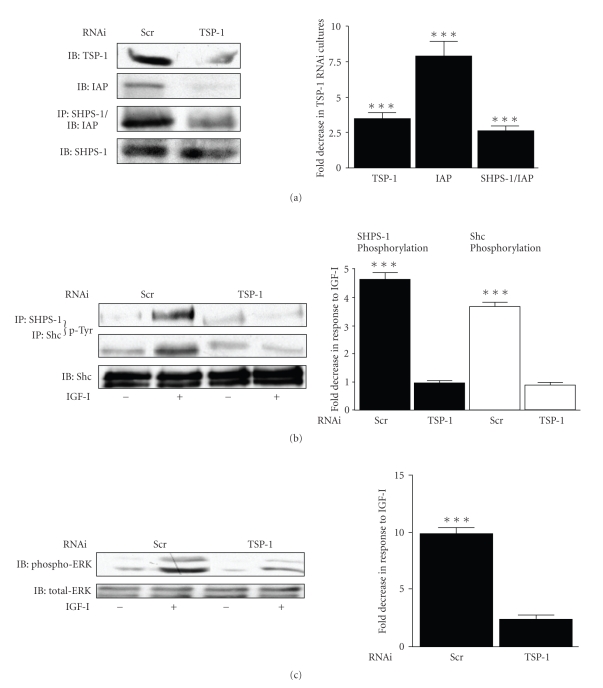
TSP-1 protein levels regulate the response of SMC to hyperglycemia. (a) SMCs expressing either the TSP-1 or a scrambled (Scr) RNAi construct were grown to confluency in DMEM containing 25 mM glucose and then incubated overnight in SFM with 25 mM. Equal amounts of lysates were either subject to immunoprecipitation (IP) followed by immunoblotting or immunoblotted (IB) directly with the antibody indicated. The graph shows the mean fold decrease of each protein in the RNAi cultures compared with the Scr control of three independent experiments, (***P < .005). (b) SMC expressing either the TSP-1 or a scrambled (Scr) RNAi construct were grown to confluency in DMEM containing 25 mM glucose and then incubated overnight in SFM with 25 mM prior to exposure to IGF-I (50 ng/mL) for 5 minutes (+). Equal amounts of lysates were then subject to immunoprecipitation (IP) followed by immunoblotting (IB) with the antibody indicated. The graph shows the mean fold increase in phosphorylation of each protein in response to IGF-I (***P < .005 when the extent of phosphorylation in the absence of IGF-I is compared to the addition of IGF-I). (c) SMC expressing either the TSP-1 or a scrambled (Scr) RNAi construct were grown to confluency in DMEM containing 25 mM glucose and then incubated overnight in SFM with 25 mM prior to exposure to IGF-I (50 ng/mL) for 5 minutes (+). Equal amounts of lysates were then subject to immunoblotting (IB) with either an antiphospho or total ERK antibody. The graph shows the mean fold increase in phosphorylation in response to IGF-I (***P < .005 when the extent of phosphorylation in the absence of IGF-I is compared to the addition of IGF-I).

In SMC expressing the TSP-1 RNAi construct, maintained in high glucose, there was a significant (3.5 ± 0.5 fold [mean ± SEM, *n* = 3, *P* < .005]) decrease in TSP-1 protein in the ECM compared with SMC expressing a scrambled RNAi construct (Figure 3(a) top panel). There was a significant (7.8 ± 1.1 fold [mean ± SEM, *n* = 3, *P* < .005]) decrease in the amount of intact CD47/IAP that could be detected compared with control (Figure 3(b) second panel). There was also a significant (2.6 ± 0.3 fold [mean ± SEM, *n* = 3, *P* < .005]) decrease in CD47/IAP association with SHPS-1 (Figure 3(b) third panel).

In SMC expressing the TSP-1 RNAi construct IGF-I stimulation of SHPS-1 and Shc phosphorylation was completely inhibited (Figure 3(b)). This was associated with an inhibition in IGF-I stimulated ERK1/2 phosphorylation (Figure 3(c)). SMC expressing the scrambled RNAi construct responded to IGF-I with significant increases in SHPS-1, Shc and ERK1/2 phosphorylation (Figures 3(b) and 3(c)).

### 3.4. Addition of Recombinant TSP-1 to the TS-1 RNAi Cultures Restored IGF-I Signaling

**Figure 4:**
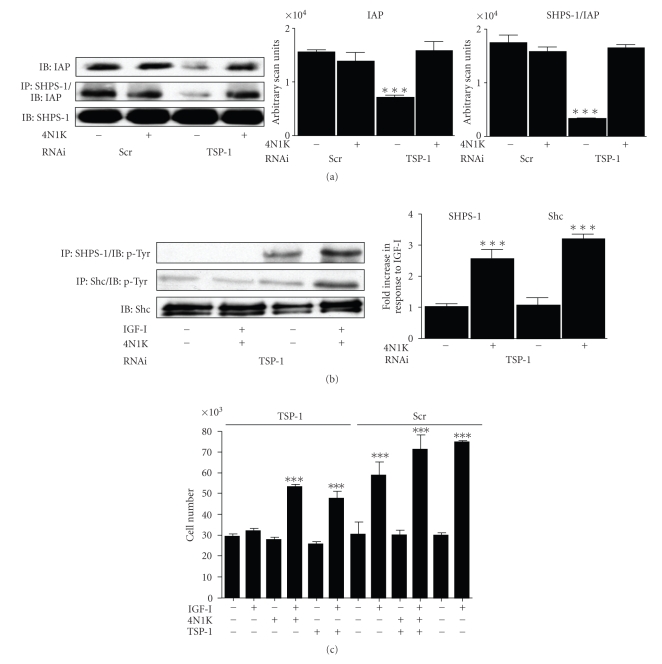
Restoration of the IGF-I response by addition of the CD47/IAP binding domain of TSP-1. (a) SMCs expressing either the TSP-1 or a scrambled (Scr) RNAi construct were grown to confluency in DMEM containing 25 mM glucose and then incubated overnight in SFM with 25 mM glucose. After overnight incubation in SFM cells were incubated for 6 hours with 4N1K (1 *μ*g/mL) prior to lysis. The amount of intact IAP in each lysate was determined by immunoblotting with the anti-IAP antibody specific for intact IAP and the association between IAP and SHPS-1 was determined by coimmunoprecipitation. The graphs shows the results derived from western immunoblots from three similar experiments expressed as arbitrary scanning units increase (***P < .005 when the amount in the TSP-1 lysate is compared with the Scr cell lysate). (b) SMCs expressing the TSP-1 RNAi construct were grown to confluency in DMEM containing 25 mM glucose and then incubated overnight in SFM with 25 mM glucose. After overnight incubation in SFM cells were incubated for 6 hours with 4N1K (1 *μ*g/mL) then treated with IGF-I (+) where indicted for 5 minutes prior to lysis. SHPS-1 and Shc phosphorylation in equal amounts of cell lysate was determined by immunoprecipitation and immunoblotting with an antiphosphotyrosine antibody (PY99). The graphs shows the results derived from western immunoblots from three similar experiments expressed as fold increase in phosphorylation after treatment with IGF-I (***P < .005 when the phosphorylation in the presence of 4N1K is compared with the absence). (c) 2 × 10^4^ cells grown 25 mM were plated in each well of a 24 well plate prior to exposure to 4N1K (1 *μ*g/mL) and IGF-I (100 ng/mL) in DMEM + 0.2% platelet poor plasma. 48 hours after the addition of IGF-I cell number was determined by trypan blue staining and counting. ***P < .005 when cell number in response to IGF-I is compared with control.

We examined the effect of adding the 4N1K peptide to the TSP-1 RNAi cultures in high glucose. Addition of 4N1K restored the amount of intact CD47/IAP that could be detected to the same level as seen in control cultures. This was associated with a corresponding increase in IAP association with SHPS-1 (Figure 4(a)).

Addition of 4N1K facilitated a 2.5 ± 0.3 fold increase in SHPS-1 in response to IGF-I in TSP-1 RNAi cultures [mean ± SEM, *n* = 3, *P* < .005] (Figure 4(b)). The increase in SHPS-1 association in the presence of 4N1K was also associated with a significant 3.2 ± 0.1 fold increase in Shc phosphorylation in response to IGF-I [mean ± SEM, *n* = 3, *P* < .005]. Consistent with the lack of signaling in response to IGF-I, TSP-1 RNAi cultures demonstrated no significant increase in cell number in response to IGF-I (Figure 4(c)). However, when TSP-1 RNAi cultures were treated with 4N1K there was a significant increase in cell number in response to IGF-I equivalent to the response noted in control cultures (Figure 4(c)).

### 3.5. TS-1 Regulation of *β*3 Phosphorylation

**Figure 5:**
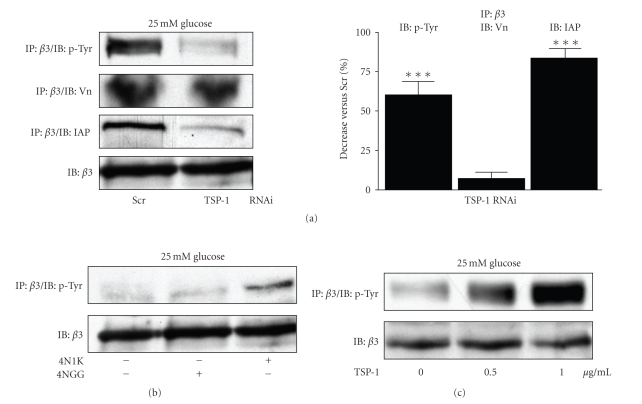
TSP-1 protein levels regulate *β*3 phosphorylation. SMCs expressing the TSP-1 or a scrambled (Scr) RNAi construct were grown to confluency in DMEM containing 25 mM glucose and then incubated overnight in SFM with 25 mM glucose prior to treatment with 4N1K or 4NGG (1 *μ*g/mL) or TSP-1 (0.5 and 1 *μ*g/mL) for 6 hours prior to lysis. The graph shows the mean fold decrease, of three independent experiments. ***P < .005 when the level in TSP-1 RNAi cultures is compared with controls.

Our previous studies have demonstrated the importance of the *α*V*β*3 integrin in regulating IGF-I signaling responses of SMC in high glucose conditions [4]. CD47/IAP was initially isolated through its association with *α*V*β*3 and has been shown by us and others to increase *α*V*β*3 ligand binding [16]. Ligand binding to *α*V*β*3 regulates *β*3 phosphorylation which we have shown is critical for subsequent downstream signaling in response to IGF-I [17]. The TSP-1 RNAi cultures (in high glucose) had a significant (60 ± 9% [mean ± SEM, *n* = 3, *P* < .005]) reduction in *β*3 phosphorylation compared with control cultures (Figure 5(a) top panel). There was no significant difference in *β*3 binding to its ligand Vn to account for this reduction in *β*3 phosphorylation (Figure 5(a) second panel). However, there was a significant (83 ± 6.4% [mean ± SEM, *n* = 3, *P* < .005]) reduction in IAP association with *β*3 in cells with reduced TSP-1 protein (Figure 5(a) third panel).

The addition of 4N1K, but not 4NGG, resulted in a significant (5.3 ± 0.5 fold [mean ± SEM, *n* = 3, *P* < .005]) increase in *β*3 phosphorylation (Figure 5(b)). Addition of full-length TSP-1 also increased *β*3 phosphorylation by 2.7 ± 0.5 fold [mean ± SEM, *n* = 3, *P* < .005].

### 3.6. Glucose Regulation of TS-1 Levels in SMC

**Figure 6:**
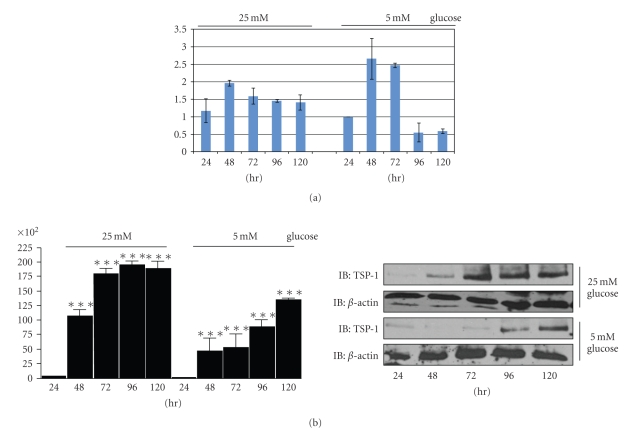
Glucose regulation of TSP-1 mRNA and protein. (a) RNA prepared from whole cell lysates. One microgram of total RNA was reverse transcribed. Two *μ*L of the cDNA was use in a RT-PCR reaction. A standard curve was generated using the 24 hour normal glucose sample (10-fold dilutions from 1 to 10,000). The amount of RNA is expressed relative to the level in the 24 hour normal glucose sample. (b) An equal number of SMCs were plated in growth medium containing 5 or 25 mM glucose. Extracellular matrix and cell lysates were prepared from parallel cultures after 24, 48, 72, 96, and 120 hours and equal amounts of protein were separated by SDS-PAGE and immunoblotted with the appropriate antibody. The graph show the results expressed as arbitrary scanning units, of three independent experiments. ***P < .005 when the level of TSP-1 protein at each time point is compared with the level at 24 hours.

Using real-time PCR we examined TSP-1 mRNA levels in SMC maintained in normal versus high glucose conditions. There was no significant difference in the amount of TSP-1 mRNA after 24 in either normal or high glucose containing medium. There was more mRNA in the normal glucose culture after 48 and 72 hours. However, after 96 and 120 hours of culture, there was more mRNA in the high glucose culture compared with the normal glucose culture (Figure 6(a)).

We examined TSP-1 protein levels in ECM preparations (containing equal amounts of total protein) from parallel time-course cultures. There was a significant increase in the amount of TSP-1 that could be detected in the high glucose cultures after 48 hours compared with 24 hours and this increased further after 72 hours and remained constant at 96 and 120 hours of culture. TSP-1 protein also accumulated in the ECM of SMC maintained in normal glucose over time but there was significantly less TSP-1 at each time point compared with the amount of TSP-1 in the high glucose cultures (Figure 6(b)).

### 3.7. TSP-1 Is Degraded More Rapidly in Normal Glucose SMC Cultures

Since the changes in TSP-1 protein over time in culture did not exactly parallel the changes in mRNA over the same time period in the two different culture conditions we considered whether there was a difference in TSP-1 degradation. 

**Figure 7:**
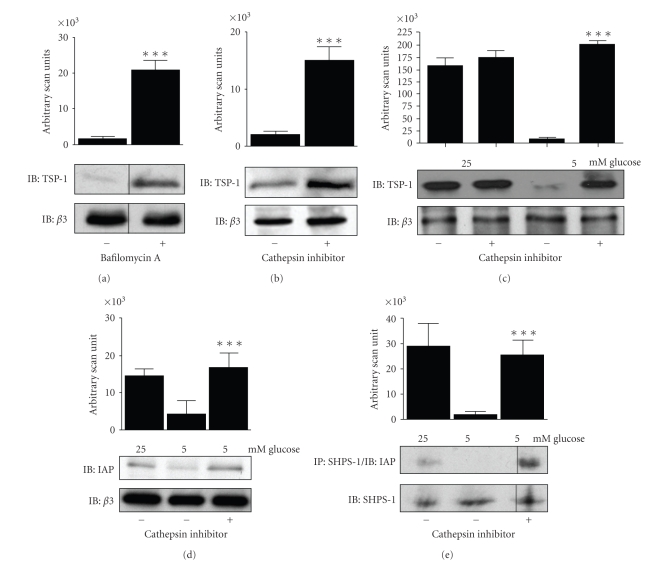
TSP-1 degradation. (a)–(e) SMCs grown to confluency in medium containing 25 or 5 mM glucose were incubated overnight in SFM and then treated with (+) a cathepsin inhibitor (100 nM), (+) bafilomycin (100 nM) or vehicle (−) for 6 hours prior to extracellular matrix preparation and immunoblotting with the anti-TSP-1 antibody or cell lysis/immunoprecipitation with the antibodies indicated. The graphs show the results of arbitrary scanning units derived from the western immunoblots of three independent experiments. Vertical line indicates where discontinuous bands from the same gel were used.

In the presence of Bafilomycin A, an inhibitor that raises the pH of lysosomes, thereby inhibiting the activity of lysosomal enzymes, there was a significant increase in TSP-1 protein in SMC cultured in normal glucose (Figure 6(a)). In the presence of a specific Cathepsin L inhibitor there was a significant increase in the amount of TSP-1 protein that could be detected in the ECM of SMC cultured in normal glucose (Figure 6(b)). Consistent with the difference in TSP-1 levels between high and normal glucose cultures being due to a difference in TSP-1 degradation there was no significant increase in TSP-1 protein levels in SMC in high glucose in the presence of the Cathepsin-L inhibitor (Figure 7(c)).

In the presence of this inhibitor, there was a significant increase in the amount of CD47/IAP that could be detected, equivalent to that detected in high glucose (Figure 7(d)). Similarly, the increase in IAP association with SHPS-1 in the presence of Cathepsin L inhibitor was equal to the level of CD47/IAP association with SHPS-1 in high glucose conditions (Figure 7(e)).

## 4. Discussion

Our previous studies in vitro [6], confirmed in vivo [9], have determined that the decrease in CD47/IAP cleavage is a key aspect of the response of SMC to IGF-I in hyperglycemic conditions. Using RNAi to reduce TSP-1 protein levels and a peptide homologous to the CD47/IAP binding site of TSP-1 we were able to demonstrate that the enhancing effect of hyperglycemia on IGF-I signaling was mediated by TSP-1 binding to CD47/IAP. Thus, the increase in CD47/IAP induced by the presence of TSP-1 leading to an increase in CD47IAP association with SHPS-1 is a potential mechanism to explain the increase in SMC proliferation and migration associated with accelerated atherosclerosis in patients with diabetes.

The mechanism by which TSP-1 may protect CD47/IAP from cleavage remains to be determined. One possibility is that TSP-1 binds to and inhibits accessibility of the CD47/IAP cleavage site. We have determined previously that the protease responsible for IAP cleavage in normal glucose conditions is the matrix metalloprotease (MMP) MMP-2 [6]. An alternative explanation for the mechanism by which TSP-1 protects IAP from cleavage is via its binding directly to MMP-2 and regulation its activity. TSP-1 was shown to bind to MMP-2 through its properdin-like type 1 repeats and inhibit activation of the MMP-2 zymogen [18]. However, given that the IAP binding site peptide alone was sufficient to protect CD47/IAP from cleavage, it seems more likely that TSP-1 is protecting CD47/IAP from cleavage by inhibiting MMP-2 access to the cleavage site.

Sajid et al. [19] have demonstrated that after balloon injury in baboons, there was a significant increase in TSP-1 in the neointima and media. This was associated with a comparable increase in CD47/IAP. Thus it seems reasonable to propose that increased cellular levels of TSP-1 and the subsequent protection of IAP from cleavage are common responses of vascular SMC to stress (e.g., mechanical injury or hyperglycemia).

While interpretation of our finding of increased TSP-1 in the aorta homogenates is limited since the homogenates contain a mixture of cell types, our finding of increased TSP-1 in aorta from hyperglycemic pigs is consistent with prior studies that detected increased levels of TSP-1 in specific areas of the blood vessels of diabetic Zucker rats [20]. Other models of diabetes have also demonstrated increases in TSP-1 in cardiac fibroblasts [21], myocytes [22, 23], and SMC [24]. Protein levels of TSP-1 are controlled at the level of RNA synthesis and stability [24, 25]. Direct stimulation of aortic smooth muscle cells with high glucose was shown to result in the transcriptional activation of the thrombospondin gene [20, 24, 25]. Our data in which TSP-1 mRNA was studied over a period of 5 days did not show a pattern of RNA changes that reflected the pattern of TSP-1 protein increase. It is interesting to note that in several previous studies [20, 24] SMC were transferred from 5 mM glucose acutely to 30 mM glucose and this experimental paradigm may have influenced the results obtained and may explain why we obtained a different pattern. 

Using inhibitors of lysosomal degradation we were able to restore TSP-1 in normal glucose to that seen in high glucose whereas we did not detect a significant increase in TSP-1 protein levels in high glucose cultures treated in a similar manner. This suggests that a significant contributor to the increase in TSP-1 protein levels in hyperglycemic conditions is due to its protection from lysosomal degradation. To our knowledge this is the first report of glucose mediated regulation of TSP-1 protein stability. In addition to the regulation of TSP-1 mRNA it has also been shown to be regulated by its rapid internalization by the endocytic receptor LRP-1 (low density lipoprotein-related protein) that leads to its degradation [26–28]. Other studies have also shown that LRP-1 levels are decreased in the aortic arch in diabetic hamsters [29] and also in the blood brain barrier of streptozotocin induced diabetic rats [30]. However, the mechanism by which glucose regulates LRP-1 has not been elucidated. If, in future studies, we can verify that LRP-1 mediates TSP-1 uptake in SMC and that this is modified by hyperglycemia we will be able to determine whether it is the levels of the receptor that differ between normal and high glucose or some other difference that accounts for the change in TSP-1 uptake and more precisely define the mechanism by which glucose regulates TSP-1 uptake and degradation.

Several biochemical pathways have been associated with hyperglycemia including diacylglycerol production, and the subsequent activation of the protein kinase C (PKC) pathway, flux through the polyol metabolic pathway, accumulation of advanced glycation end products (AGE) and cytokine secretion [31]. The production of excess reactive oxygen species has been suggested to be the causal link between hyperglycemia and these changes [31]. Since, at this point we do not know the receptor that mediates TSP-1 uptake it is difficult to predict which specific glucose mediated event reduces TSP-1 uptake in SMC. Interestingly however, it has been shown in cells from glioblastoma tumors that activation of PKC (alpha) resulted in the down-regulated LRP [32], suggesting a possible relationship between the activation of the PKC pathway, down-regulation of LRP and thus decreased cellular uptake of TSP-1. Additionally, it has been shown that there are changes in the relative distribution of the receptor for advanced glycation end products (RAGE) and LRP in the blood brain barrier of the human hippocampus of patients with Alzheimer's disease; as levels of RAGE increase then levels of LRP are decreased [33]. While merely speculative these findings provide potential directions to pursue that may ultimately shed light on the precise mechanism by which TSP-1 cellular uptake and degradation are reduced in SMC maintained in hyperglycemia.

CD47/IAP was identified through its association with *α*V*β*3 [16]. Furthermore, CD47/IAP binding to *α*V*β*3 has been shown to increase integrin clustering [34]. Importantly, the extracellular domain of CD47/IAP, which is the region of CD47/IAP that is lost during cleavage, is critical for its ability to induce *α*V*β*3 clustering [34]. TSP-1 binding to CD47/IAP has been implicated in stimulating a high affinity state in *α*IIb*β*3 leading to activation of the integrin and platelet spreading, stimulation of platelet aggregation [35, 36]. Our study suggests that the TSP-1 increase in CD47/IAP plays a dual role in regulating the response of SMC to IGF-I in hyperglycemic conditions. In addition to increasing CD47/IAP association with SHPS-1 it also regulates CD47/IAP association with *α*V*β*3 thereby regulating the activation status of the integrin, reflected in *β*3 phosphorylation. We have shown previously, by expressing *β*3 in which both tyrosine residues in the cytoplasmic domain were mutated to phenylalanine, that the phosphorylation of *β*3 is required for its positive effect on IGF-I signaling [17]. 

The results from this study demonstrate that glucose regulation of TSP-1, by reducing its lysosomal degradation, enhances IGF-I signaling in SMC by protecting CD47/IAP from cleavage thereby increasing its association with its ligands SHPS-1 and *α*V*β*3. These results support the hypothesis that the glucose stimulated increase in TSP-1 may play an important role in the accelerated atherosclerosis associated with diabetes and identifying the receptors that mediate the effects of TSP-1 is a potential strategy to prevent the initiation and development of atherosclerotic lesions in these patients.
